# The effect of genetic structure on molecular dating and tests for temporal signal

**DOI:** 10.1111/2041-210X.12466

**Published:** 2015-09-22

**Authors:** Gemma G. R. Murray, Fang Wang, Ewan M. Harrison, Gavin K. Paterson, Alison E. Mather, Simon R. Harris, Mark A. Holmes, Andrew Rambaut, John J. Welch

**Affiliations:** ^1^ Department of GeneticsUniversity of CambridgeDowning StreetCambridge CB2 3EHUK; ^2^ Department of Veterinary MedicineUniversity of CambridgeMadingley RoadCambridge CB3 0ESUK; ^3^ School of Biological, Biomedical and Environmental SciencesUniversity of HullCottingham RoadHull HU6 7RXUK; ^4^ Wellcome Trust Sanger InstituteHinxton CB10 1SAUK; ^5^ Institute of Evolutionary BiologyUniversity of EdinburghKing's BuildingsEdinburgh EH9 3FLUK

**Keywords:** Bayesian dating, dated‐tips, pathogen origins, permutation tests, *Staphylococcus aureus*

## Abstract

1. ‘Dated‐tip’ methods of molecular dating use DNA sequences sampled at different times, to estimate the age of their most recent common ancestor. Several tests of ‘temporal signal’ are available to determine whether data sets are suitable for such analysis. However, it remains unclear whether these tests are reliable.
2. We investigate the performance of several tests of temporal signal, including some recently suggested modifications. We use simulated data (where the true evolutionary history is known), and whole genomes of methicillin‐resistant *Staphylococcus aureus* (to show how particular problems arise with real‐world data sets).
3. We show that all of the standard tests of temporal signal are seriously misleading for data where temporal and genetic structures are confounded (i.e. where closely related sequences are more likely to have been sampled at similar times). This is not an artefact of genetic structure or tree shape *per se*, and can arise even when sequences have measurably evolved during the sampling period. More positively, we show that a ‘clustered permutation’ approach introduced by Duchêne *et al*. (*Molecular Biology and Evolution*,** 32**, 2015, 1895) can successfully correct for this artefact in all cases and introduce techniques for implementing this method with real data sets.
4. The confounding of temporal and genetic structures may be difficult to avoid in practice, particularly for outbreaks of infectious disease, or when using ancient DNA. Therefore, we recommend the use of ‘clustered permutation’ for all analyses. The failure of the standard tests may explain why different methods of dating pathogen origins have reached such wildly different conclusions.

## Introduction

Molecular dating uses evolutionary change between homologous DNA sequences to infer the time since their most recent common ancestor (*t*_MRCA_). If the genomes were sampled at similar times, then this inference requires external temporal information, such as a known rate of evolution, to calibrate the molecular clock. But if the genomes were sampled at sufficiently different times, then the sampling dates are all the temporal information required (Rambaut 2000; Drummond, Pybus & Rambaut 2003b; Drummond *et al*. 2003a). Such ‘dated‐tip’ methods have been particularly useful in the study of viral and bacterial pathogens and have been used to understand the origins and spread of diseases, as well as transmission pathways within a single outbreak (e.g. Smith *et al*. 2009; Didelot *et al*. 2012; McAdam *et al*. 2012; Gire *et al*. 2014).

Dated‐tip methods are only valid if there is temporal signal in the data. This will not be the case if the sampling period was too short for sufficient evolutionary change to occur or if evolutionary rates were too variable (Drummond, Pybus & Rambaut 2003b; Firth *et al*. 2010; Duchêne *et al*. 2015a). However, evolutionary rates are often unknown, and molecular dating methods will usually converge on an estimate whether or not temporal signal is present (Firth *et al*. 2010). As such, it is crucial to test the molecular data for temporal signal.

Several approaches have been used to test for temporal signal. The simplest is a linear regression of phylogenetic root‐to‐tip distance against sampling date (Buonagurio *et al*. 1986; Shankarappa *et al*. 1999; Korber *et al*. 2000; Drummond, Pybus & Rambaut 2003b). If sampling dates are sufficiently different, then more recently sampled sequences will have undergone substantially more evolutionary change than earlier sampled sequences, and this should create a strong positive correlation. This test obviously requires a rooted phylogeny, and when the root is unknown, it is common to estimate the root simultaneously with the regression, so as to maximize the model fit (Drummond *et al*. 2003a). Significance is not generally calculated, because root‐to‐tip distances are non‐independent, but Navascués, Depaulis & Emerson (2010) suggest using permutation, asking whether the correlation is stronger than expected if the sampling dates were assigned to sequences at random.

Linear regression is a crude method of molecular dating, but analogous tests can be used with more formal methods. Most commonly, the *t*_MRCA_ or rate estimate from a Bayesian dated‐tip analysis is used as the test statistic. If more recently sampled sequences have undergone more molecular evolution, then the true sampling dates should yield a *t*_MRCA_ that differs substantially from the equivalent estimates with the sampling dates randomly permuted over sequences (Ramsden, Holmes & Charleston 2009; e.g. Duffy & Holmes 2009; Firth *et al*. 2010; Fraile *et al*. 2011; Pagán & Holguín 2013; Duchêne, Holmes & Ho 2014b; Duchêne *et al*. 2015a).

Finally, a distinct approach uses model selection and compares the fit of models with the sampling dates included or excluded, thereby failing to take special account for any evolution that might have taken place during the sampling period (Rambaut 2000; Drummond, Pybus & Rambaut 2003b; Drummond *et al*. 2003a; Baele *et al*. 2012). Temporal signal is confirmed if the inclusion of the sampling dates improves the fit.

All of the approaches above are widely used, but it is not clear how well they identify temporal signal, especially if we define temporal signal as the ability of a data set to yield reliable date estimates. Previous studies have shown that dated‐tip methods can be unreliable not only for data with too short a sampling period or too variable an evolutionary rate, but also for data with strong population structure (Navascués & Emerson 2009) or imbalanced trees (Duchêne, Duchêne & Ho 2015b). Furthermore, Duchêne *et al*. (2015a) showed that the Bayesian permutation test gave false evidence of temporal signal for simulated data where the sampling period was too short, but where clusters of closely related sequences were sampled at the same time, that is where temporal and genetic structures were confounded. To solve this problem, they introduced a ‘clustered permutation approach’ where dates were randomly reassigned among clusters of sequences sampled on the same date.

Here, we investigate the performance of tests of temporal signal on a variety of simulated and real‐world structured data sets. We show that while structured data can generate accurate estimates of the *t*_MRCA_ with dated‐tip methods, when temporal and genetic structures are confounded, estimates are consistently misleading, regardless of the level of temporal structure in the data. We further show that the standard tests of temporal signal fail to identify data sets that result in unreliable estimates when temporal and genetic structures are confounded. We demonstrate that the clustered permutation approach of Duchêne *et al*. (2015a) can be applied to both the regression and Bayesian tests for temporal signal, and that it successfully identifies those data sets that give reliable estimates in the presence of confounded genetic structure. Finally, through analysis of two sets of whole‐genome data from *Staphylococcus aureus*, with very different sampling periods, we develop methods of applying these tests to real data and show that confounding can arise naturally from clinical sampling practice, suggesting that the unreliable date estimates may be widespread.

## Materials and methods

### Data Sets

Details of our simulated and real data sets are provided in the Supporting information.

### Basic Dating Analyses

We estimated the *t*_MRCA_ for all of our data sets using beast v1.8 (Drummond *et al*. 2012). In all cases, we used a constant population size coalescent prior for the node ages, and (except for Bayes factor calculations) the BEAUti v1.8 default priors for all other parameters (Drummond *et al*. 2012). After each run, convergence was assessed using tracer v1.6 (Rambaut *et al*. 2014) and burn‐in removed as required. For the *t*_MRCA_, we recorded the maximum *a posteriori* (MAP) estimate, estimated from the MCMC using the Venter mode estimator from the r package *modeest* (Venter 1967; Poncet 2012), and the 95% highest posterior density (HPD) interval.

For the simulated data sets, we fit the same evolutionary model that was used to simulate the data, namely the HKY+Γ substitution model and a strict molecular clock. For the reanalysis of the *S. aureus* data, we also used the HKY+Γ substitution model. For the data from Holden *et al*. (2013), we used the uncorrelated log‐normal relaxed molecular clock (replicating the published analysis), whereas for the data from Paterson *et al*. (2015) we used a strict clock due to the small number of variable sites.

### Tests of Temporal Signal

#### Regression test

To regress phylogenetic root‐to‐tip distance against sampling date, we obtained crude root‐to‐tip distances from a neighbour‐joining tree estimated using a K80 nucleotide substitution model with the ape package in r (Paradis, Claude & Strimmer 2004). Following the path‐o‐gen software (Rambaut 2013), the root was fit simultaneously with the regression, so as to minimize the residual mean squares (see also Korber *et al*. 2000). Following the suggestion of Navascués, Depaulis & Emerson (2010), the significance of the regression was assessed by random permutation of the sampling dates over the sequences, using the correlation coefficient as the test statistic. For all reported results, we generated 1000 replicates of the data, with the sampling dates randomly permuted. The *P*‐value is the proportion of replicates with a test statistic greater than or equal to the true value. The null hypothesis is that a negligible amount of evolution took place between the sampling dates, so that the correlation observed can be attributed to stochastic variation in molecular branch length estimates and (when the root is not known independently) to our having rooted the tree to maximize clocklikeness.

#### Bayesian dating permutation tests

To test for temporal signal using Bayesian dating, each analysis was repeated 10 times, after randomly permuting the sampling dates across sequences (e.g. Ramsden, Holmes & Charleston 2009). We then asked whether the *t*_MRCA_ estimate from the true data was outlying when compared with the estimates from the randomly permuted data. This is not standard hypothesis test, since each of the 11 estimates is associated with uncertainty, and therefore, *P‐*values were not calculated. We note that the choice of the *t*_MRCA_ as a test statistic is somewhat arbitrary, and that other alternatives (such as mean rate) could also be used. Unless one or other statistic was of particular interest, statistics might be preferred whose posterior distributions are easier to estimate from the MCMC.

#### Clustered permutation tests

**Figure 1:**
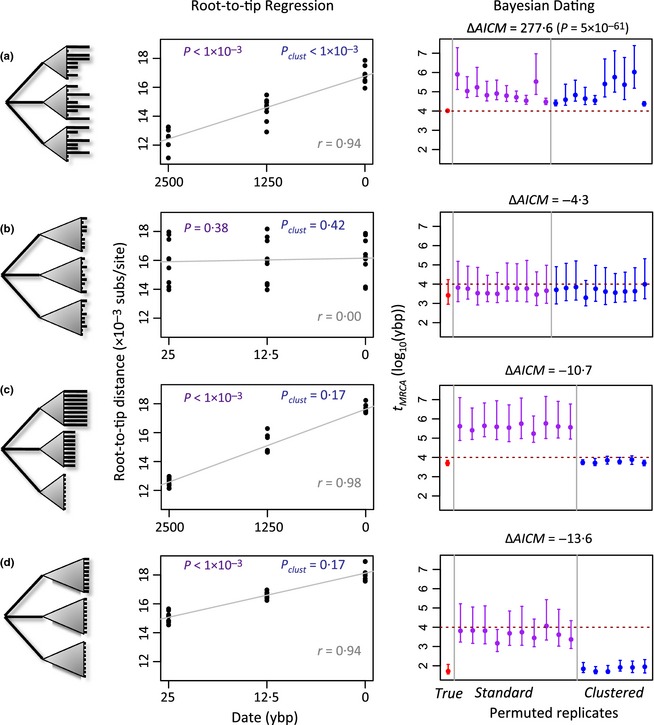
The left‐hand column shows schematic representations of the tree topologies over which evolution was simulated. The grey triangle represents the variable branching patterns of a simulated coalescence process. The middle column shows results of the regression of root‐to‐tip distance against sampling date. A significant positive correlation is consistent with the presence of temporal signal. *P*‐values were obtained by random permutation of sampling dates across sequences (*P*) or monophyletic clusters of sequences that shared a sampling date (*P*_clust_). The right‐hand column shows the maximum *a posteriori* estimate of the *t*_MRCA_ with 95% highest posterior density intervals (red) as inferred using beast. These are compared to equivalent estimates from data sets with the sampling dates randomly permuted across sequences (purple), or clusters of sequences (blue). For the model selection approach, we report the increase in AICM values when sampling dates were included in the analysis. (a) and (b) represent a ‘balanced’ sampling strategy where each clade was sampled equally thoroughly at each of the sampling times; (c) and (d) represent a confounded sampling strategy where each clade was sampled at a different time. For (a) and (c), true temporal structure is high, such that a substantial amount of molecular evolution could occur between the sampling dates, while for (b) and (d), temporal structure is low.

The permutation tests described above assume that the sampling dates are exchangeable under the null. This will not be true if closely related sequences were preferentially sampled at the same date. A heuristic approach to dealing with this artefact was introduced by Duchêne *et al*. (2015a). Their approach is to randomize dates over clusters of sequences, rather than individual sequences. Clusters are defined as monophyletic clades, which were sampled at the same time. If we have *n* clusters, then the maximum number of permutations of these clusters is *n*!, and if each sampling date is associated in more than one of the clusters, then the total number of unique permutations is $n!\prod_{i}^{\mathrm{dates}}m_{i}^{-1}$ where *m*_*i*_ is the number of clusters associated with sampling date *i*. When this number is suitably small, it is easiest to generate all possible permutations. For example, in Fig. 1c,d, there were only 3! = 6 possible permutations, which made 1/6 the smallest possible *P‐*value for these extreme cases. For the simulated data, we identified single‐date clusters from a neighbour‐joining tree, rooted to minimize the residual mean squares of a linear regression of sampling time against root‐to‐tip distance (see Results for definitions for real data). All tests were implemented in r scripts (R Core Team 2014), which are provided in the Supporting information.

#### Tests of model fit

A final test of temporal signal is to compare some measure of model fit for phylogenetic analyses with or without sampling dates (Baele *et al*. 2012). In practice, for the ‘no dates’ model, to keep the two cases as similar as possible, we set all sequences to the most recent sampling date in the original data set. We compared two model comparison statistics. The AICM is computationally cheap and robust to specification of improper priors. It can also be transformed into a true hypothesis test, using Akaike weights (Burnham & Anderson 2002). To do this, when the ‘with dates’ model was preferred, the relative support for this model, equivalent to the *P*‐value, was calculated as $w={\left(1+e^{\frac{1}{2}\Delta \mathrm{AICM}}\right)}^{-1}$, where ΔAICM is the improvement in the fit. AICM was estimated in tracer v1.6 (Rambaut *et al*. 2014), which was also used to check convergence.

To calculate Bayes factors, we used the path sampling approach with 100 steps, as implemented in beast v.2 (Baele *et al*. 2012; Bouckaert *et al*. 2014). The method relies on the specification of priors that are proper (integrating to unity), and not too diffuse (Baele *et al*. 2012). (This may be difficult for data sets where *a priori* plausible date or rate estimates span several orders of magnitude.) We set the mean rate prior to a gamma distribution with a shape parameter of 0·1 and a scale parameter of 1, the standard deviation of the rate prior to an exponential distribution with a mean of 1, the population size prior to an exponential distribution with a mean of 100, the HKY transition–transversion parameter prior to a gamma distribution with a shape parameter of 2 and a scale parameter of 1, and the between‐site rate gamma shape prior to an exponential distribution of mean 1.

## Results

### Dating Artefacts with Simulated Data

To illustrate the performance of tests of temporal signal on genetically structured samples, we simulated molecular data sampled on three different dates, from a highly structured population, consisting of three distinct and equally related clades, whose most recent common ancestor lived 10 000 years before the present (ybp), evolving at a comparable rate to some bacteria and viruses (1·6 × 10^−6^ subs per site per year). We applied the standard tests of temporal signal to these simulated data and estimated their *t*_MRCA_ (Fig. 1).

We first simulated data with a high degree of temporal structure, by selecting three sampling dates such that an average of 20 nucleotide substitutions per genome occurred between each sampling. We also assumed a ‘balanced’ sampling scheme, such that all three genetic clades were sampled equally thoroughly on all three dates. With this high temporal structure, and balanced sampling, the dating was a success. When correlating root‐to‐tip distance with sampling dates, all of the 1000 simulated data sets showed the signature of temporal signal (see Fig. S1a for a histogram of *r*‐values). Figure 1a shows a detailed analysis of a single typical replicate, with a permutation test, confirming that the correlation was unlikely to have arisen by chance (Fig. 1a, middle column; see also Table S1); indeed, for these simulated data, variation around the regression line must be attributed to stochastic variation in the substitution process, or to estimation error in the branch lengths. The intercept of the regression was also similar to the true *t*_MRCA_ used to simulate the data. Bayesian molecular dating with beast (Drummond *et al*. 2012) also performed well (Fig. 1a, right‐hand column): the *t*_MRCA_ estimate (red point) was accurate and precise, and also highly outlying when compared with replicate analyses with sampling dates randomly permuted (purple points).

We next simulated data with the same balanced sampling, but little temporal structure, that is with sampling dates that were so close that only 0·2 substitutions per genome were expected between them (Fig. 1b). In this case, *t*_MRCA_ estimates were highly inaccurate, but tests of temporal signal correctly indicated that these estimates could not be trusted. In particular, none of the 1000 data sets gave high *r*‐values (Fig. S1b), and tests confirmed that similar results could be obtained after randomly permuting the sampling dates. Therefore, with balanced sampling (Fig. 1a,b), tests of temporal signal perform well.

Performance declined substantially when sampling was confounded with genetic structure, that is when each genetic clade was sampled on a different date (Fig. 1c,d). In these cases, estimates of the *t*_MRCA_ were highly inaccurate, but tests of temporal signal wrongly indicated that the inaccurate dates could be trusted. These artefacts occurred both when there was high temporal structure (Fig. 1c), and when there was low temporal structure (Fig. 1d). Indeed, with low temporal structure, over a third of the simulated data sets showed a high correlation between sampling date and root‐to‐tip distance (Fig. S1d), and a typical data set gave strong evidence of temporal signal, despite yielding a wildly inaccurate estimate of the *t*_MRCA_: 51 ybp, as opposed to the true value of 10 000 ybp.

**Figure 2:**
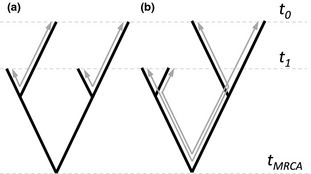
Illustrative phylogenies in which genetic and temporal structure are (a) unconfounded or (b) confounded. Grey arrows describe the distance between pairs of sequences sampled on different dates (*t*_0_ and *t*_1_).

To show why confounding misleads molecular dating, Fig. 2 illustrates two sections of phylogeny with the same sampling period, but different levels of confounding. (a) will tend to give better results than (b) for two connected reasons. First, in (b) the sampling period constitutes a much smaller proportion of the lineages that connect sequences sampled at different times. Secondly, (a) contains two quasi‐independent opportunities to measure the evolutionary change between the sampling dates, while in (b), the measurements are clearly non‐independent. As such, Fig. 2 suggests that the failure of random permutation can be understood, intuitively, as an inflation of the true sample size, when there is confounding. For this reason, one way to correct for the artefact is to identify genetic clusters (monophyletic groups) in the data that share a sampling date, and then permute dates over these clusters, rather than over the individual sequences (Duchêne *et al*. 2015a). With this approach (a) would contain four clusters, but (b) would contain only two, and so a clustered permutation test will be less likely to reach significance.

When Duchêne *et al*.'s (2015a) method of clustered permutation was applied to our simulated data, performance remained good with balanced sampling (Fig. 1a,b: *P*_clust_ for regression, blue points for the Bayesian analysis) and improved dramatically with confounded sampling (Fig. 1c,d). In particular, with confounded sampling, neither test of temporal signal reached significance, indicating – correctly – that both date estimates were unreliable.

Clustered permutation corrects for the confounding of genetic and temporal structure, but sometimes this confounding can arise from the evolutionary process itself, and not from sampling artefacts. Any evolutionary change in the genetic constitution of a population could lead to sequences sampled on the same date being more closely related to each other. A classic example is the ‘ladderized’ genealogy of influenza A, caused by regular selective sweeps (Grenfell *et al*. 2004), but the same effect could arise from genetic drift (Gray, Pybus & Salemi 2011). In either case, temporal and genetic structures are inherently confounded, and so clustered permutation becomes conservative. To explore the power of the clustered tests in this situation, we simulated ladderized genealogies with high temporal structure (Fig. S2). Results showed that the clustered permutation approach was still able to detect the temporal signal (an appreciable rate of false negatives arose only when the basal clade was monophyletic, and fewer than four clusters were simulated).

### Dating Artefacts with Whole Genomes of Methicillin‐Resistant *s. aureus*

Figure 1 illustrates dating artefacts with extreme cases, but the same artefacts occur with more realistic data. In the Supporting information, we demonstrate this with simulations (Tables S1–S3, Figs S1 and S3), but it can also be observed with real‐world data.

**Figure 3:**
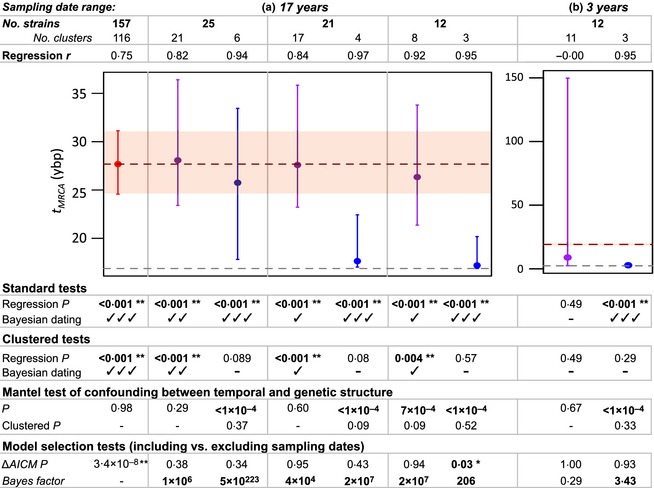
Dating analyses for *Staphylococcus aureus* genomes sampled over 17 years. Plots show the maximum *a posteriori* (MAP) estimates of the *t*_MRCA_, with 95% highest posterior density (HPD) intervals. (a) shows the estimate from the complete data set (red), and from random (purple) or confounded (blue) subsamples, all with the same common ancestor and range of sampling dates. (b) shows estimates from subsamples with a narrower sampling range, and a different true *t*_MRCA_. Red dashed lines and shaded areas describe the best estimate of the *t*_MRCA_ and its 95% HPD interval as inferred from the complete data set. Grey dashed lines show the youngest possible *t*_MRCA_, as determined by the oldest sample. Below are the results of tests of temporal signal and confounding. For beast permutation tests: ✓ indicates that the true MAP estimate lay outside of the range of the MAP estimates from the randomized data sets, ✓✓ indicates that the true MAP estimate is not within the HPD intervals of the estimates from randomized data sets, and ✓✓✓ indicates that the HPD interval of the true estimate does not overlap with the HPD intervals of estimates from the randomized data sets. For the model selection approaches, we report the probability that the model without sampling dates is the ‘true’ model (AICM analysis), or the Bayes factor support for the inclusion of sampling dates (Kass & Raftery 1995). Tests indicating temporal signal are in bold; *P <* *0·05; **P <* *0·01.

To see this, we reanalysed 157 complete genomes of epidemic methicillin‐resistant *S. aureus* sequence type (ST) 22, sampled over a 17‐year period (Holden *et al*. 2013). In agreement with Holden *et al*. (2013), we estimated the *t*_MRCA_ of these sequences as 1980, 28 years prior to the youngest sample (Fig. S4). Several lines of evidence suggest that this *t*_MRCA_ is plausible. First, all tests indicated very strong temporal signal (Fig. 3, Table S4); secondly, the inferred rate of evolution is consistent with previous estimates from *S. aureus* (Weinert *et al*. 2012); and finally, this dating places the acquisition of fluoroquinolone resistance at the time and location where fluoroquinolone drugs were first tested in UK clinical trials (Holden *et al*. 2013).

We next re‐estimated the *t*_MRCA_ after subsampling the *S. aureus* strains. These subsamples were chosen to transect the same root node and to retain the 17‐year sampling period (illustrated in Fig. S4a–f). With these constraints, we chose strains either at random (reproducing the ‘balanced’ sampling of Fig. 1a,b) or in clusters sampled in the same year (reproducing the ‘confounded’ sampling of Fig. 1c,d). In all cases, the balanced subsampling provided consistent estimates of the *t*_MRCA_ (Fig. 3a, purple points), albeit with wider credible intervals, reflecting the reduced sample size. However, the confounded subsamples produced much younger dates (Fig. 3a, blue points). In addition, all six subsamples gave evidence of temporal signal using the standard tests. If we were to trust these standard tests, we might draw quite different conclusions about the evolution of antibiotic resistance in the UK.

The same applies when we analysed subsamples collected over a 3‐year period (Fig. S4g,h). Given evolutionary rates for these strains, fewer than 7 nucleotide substitutions per genome would be expected during this entire sampling period, and so this produces ‘low temporal structure’ data sets. For both data sets, the estimated *t*_MRCA_ differed from its true value (as inferred from the complete data set; Fig. 3b, red dashed line). For the balanced subsample, all tests confirmed this lack of temporal signal, but the standard tests failed for the confounded subsample, resulting in false confidence in an inaccurate and deceptively precise estimate of the *t*_MRCA_ (Fig. 3b).

As with the simulated data, these problems can be solved by using the clustered permutation approach of Duchêne *et al*. (2015a). If we define clusters as monophyletic groups sampled in same year, regression and Bayesian approaches both correctly identified the data sets that yielded inaccurate estimates of the *t*_MRCA_. However, for these data, there is something arbitrary about the choice to cluster by year (we might also have chosen to cluster by month). This highlights the need for a test of confounding that can be applied to real‐world data sets. An obvious choice is a Mantel test of the correlation between pairwise genetic distances and absolute differences in sampling dates. Applying this test to the *S. aureus* data successfully identified the confounding in all of the confounded data sets, and in one of the smallest balanced data sets (Fig. 3). We then repeated the Mantel test after clustering the data (using the average pairwise genetic between clusters, and the absolute difference between sampling years). This test confirmed that our choice to cluster sampling dates by year was sufficiently coarse‐grained to eliminate the signal of confounding in these data (Fig. 3).

### Model Selection Approach

A test for temporal signal not considered so far, is to compare the fit of models with the sampling dates either included (‘with dates’) or ignored (‘no dates’) (Rambaut 2000; Drummond, Pybus & Rambaut 2003b; Drummond *et al*. 2003a; Baele *et al*. 2012). Various measures and estimators of model fit are available (Rambaut 2000; Suchard, Weiss & Sinsheimer 2003; Kitchen, Miyamoto & Mulligan 2008; Baele *et al*. 2012). We initially tried the AICM, an analogue of the Akaike Information Criterion, which is estimated from the MCMC (Raftery *et al*. 2007; Baele *et al*. 2012).

On simulated data, the AICM approach performed very well, showing strong support for the ‘with dates’ model whenever the *t*_MRCA_ was well estimated, and weak or no support when the *t*_MRCA_ was poorly estimated (Fig. 1, Table S1). However, for the real *S. aureus* data, only one subsample gave evidence of temporal signal, and this was a confounded subsample where the *t*_MRCA_ estimate was extremely poor (Fig. 3; Table S4). We next calculated full Bayes factors, using path sampling (Baele *et al*. 2012; Bouckaert *et al*. 2014; Leaché *et al*. 2014). This had the opposite problem: all but one subsample yielded strong support for the ‘with dates’ model. As such, model selection led to false confidence in inaccurate estimates of the *t*_MRCA_.

The failure of this approach is initially surprising, since it makes no explicit assumptions about random sampling or exchangeability. Since the approach worked well on simulated data (which used a strict clock and known substitution process), this is probably explained by model inadequacy. Evolutionary models may be good enough to provide accurate estimates of the *t*_MRCA_ *,* and yet sufficiently different from reality to render unreliable a comparison of model fit with and without sampling dates. It is also notable that the Bayes factor approach worked well when sampling was random, but not when sampling was confounded (Fig. 3; Table S4). This might be a failure analogous to ‘overfitting’, given the reduction in effective sample sizes in the confounded data sets (Fig. 2).

### Application to Data from a Single Outbreak

Examples above used data that were subsampled in a contrived way, but the same artefacts can arise with complete data sets. To illustrate this, we analysed whole genomes of *S. aureus* ST22, from a single disease outbreak. These samples were obtained from a veterinary hospital over approximately 2 months, initially from a dog admitted to the clinic (141 isolates), and then from a staff member (34 isolates) involved in the dog's treatment (Paterson *et al*. 2015).

**Figure 4:**
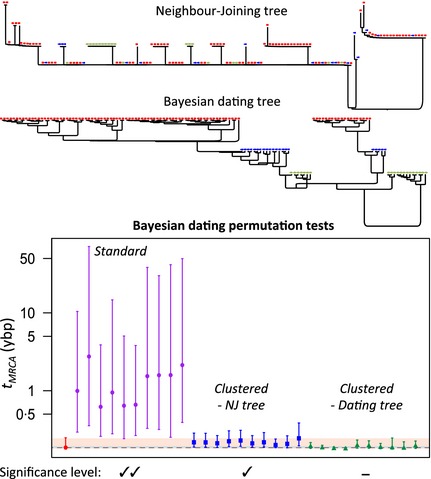
The Bayesian dating test for *Staphylococcus aureus* strains sampled from a dog during an outbreak in a veterinary hospital. Differences in the degree of clustering with sampling date are apparent between the phylogenies estimated with (the MCC tree from the Bayesian dated‐tip analysis) and without the use of temporal information (a neighbour‐joining tree). Colour and symbol shape represent strains sampled on the same date. The plot shows the maximum *a posteriori* estimates of the *t*_MRCA_ (on a log scale) with 95% highest posterior density intervals. The true estimate (red) is compared to estimates with the sampling dates randomly permuted across sequences (purple), or across single‐date clusters identified from the neighbour‐joining tree (blue), or the MCC tree (green). The blue horizontal line indicates the date of admission of the dog into the veterinary hospital. Significance levels are described in the legend of Fig. 3.

Dated‐tip analyses of these data placed the *t*_MRCA_ of the dog strains on the day after the dog's admission to the hospital, and the closely related strains from the staff member at *c*. 12 weeks earlier (Fig. 4, for the dog samples, and Fig. S5, for the staff member samples, red points). Together, these estimates suggest a scenario in which the dog was infected in the hospital, possibly by a staff member with a long‐standing infection, and where transmission was likely associated with a strong bottleneck (since all of the genetic variation in the dog can be traced back to a single feasible transmission event).

Standard tests of temporal signal supported this scenario. For the dog samples, Mantel tests yielded no evidence of confounding of temporal and genetic structures (*P *=* *0·88), and permutation tests of the Bayesian dates detected temporal signal (Fig. 4 purple points) even, weakly, with clustered permutation (Fig. 4, blue points). However, a combined analysis of all 175 isolates shows that this *t*_MRCA_ estimate – and thus the epidemiological inference – is probably unreliable. In particular, the genealogies of the dog and staff samples are intermingled, implying that they share a most recent common ancestor (Fig. S6; Paterson *et al*. 2015).

What is wrong with the analysis above? The answer is clear from comparing a neighbour‐joining tree to the Maximum Clade Consensus (MCC) tree from the beast analyses (Fig. 4). The neighbour‐joining tree has very little resolution reflecting the low genetic diversity in these data and confirms that the level of confounding is weak. In contrast, the beast tree is fully resolved and contains very high levels of confounding (Mantel tests using patristic distances: *P *< 0·001; Fig. 4, and Table S5). This shows that, in the absence of phylogenetic signal, the dating algorithm has enhanced the confounding, clustering the sequences by date to improve the fit of its clock model. (We note that no such difference was found in data sets analysed in earlier sections, where the data contained much higher levels of genetic diversity.)

It is important to note that low levels of genetic diversity would not be a problem, were there not also some genuine confounding of temporal and genetic structures, for in the absence of any confounding, a random permutation approach would succeed. For these *S. aureus* data, weak confounding – undetected by the Mantel test – probably arose from the clinical sampling practice. In particular, different sets of anatomical sites of the dog were sampled on different dates (in part, as a consequence of the progression of the disease), and genetic structure was associated with these sites (Paterson *et al*. 2015). As a result, we find genetic structure between the earliest dog samples, and those taken on later dates (permutation test of Hudson's *F*_st_ estimator: *P *<* *0·001; Hudson, Slatkin & Maddison 1992), although not between the two later dates.

When phylogenetic resolution is low, there are two ways to test for temporal signal, which avoid the artefact described above. The first is to use the regression approach, with a phylogeny that was inferred without making any assumptions about molecular rates. The second is to use the clustered Bayesian dating permutation approach, but with clusters identified from the MCC tree (Fig. 4, green points). Both approaches found no temporal signal in our *S. aureus* data (from either the dog, or the staff member; Fig. S5), confirming that *t*_MRCA_ estimates from these data cannot be trusted.

## Discussion

Molecular dates obtained with ‘dated‐tip’ methods are reliable only if the sequence data exhibit temporal signal. As such, we cannot trust dates obtained from these methods unless we can also trust the tests for temporal signal.

We have shown that all of the standard tests of temporal signal can be severely misled for data sets where temporal and genetic structures are confounded, that is when closely related sequences are more likely to have been sampled at similar times. Our results show that the reliability of date estimates cannot be determined from the degree of genetic structure *per se* (data sets in Fig. 1a–d had equally high levels of structure), nor from the number of sequences sampled (Fig. 3a shows that subsamples of any size can yield both inaccurate and accurate estimates) and nor from the overall range of the sampling dates, or level of temporal structure (which was held constant across both Figs 1a,c and 3a). However, we have shown that when confounding is present, the clustered permutation approach of Duchêne *et al*. (2015a), can give good results, whether applied to linear regression or Bayesian dating, and to data with or without temporal structure.

We have also introduced some refinements to the approach of Duchêne *et al*. (2015a), which show how clustered permutation can be best applied to real‐world data. In particular, we have shown how a Mantel test, comparing genetic distance and difference in sampling dates, can identify data sets where confounding is present (Fig. 3). We have also shown how the same test can confirm whether a particular choice of clusters has successfully removed the confounding (this is particularly useful when samples were taken on a very large range of dates, as in the *S. aureus* data from Holden *et al*. (2013). Finally, we have shown that an additional problem can arise for data with low levels of phylogenetic resolution, when dating algorithms may enhance the true level of confounding. We have suggested that, to mitigate this problem, clusters should be chosen from the tree estimated in the dating analysis (Fig. 4).

The problem of confounding, discussed here, may explain some previously noted failures of the dated‐tip approach. For example, Navascués & Emerson (2009) showed that inaccurate estimates of the *t*_MRCA_ could be obtained in structured populations when ancient and modern sequences came from different genetic clusters. Indeed, confounding is likely to be particularly severe when the temporal information comes from a small number of ancient DNA sequences. Similarly, Duchêne, Duchêne & Ho (2015b) showed that inaccurate results could be obtained when trees were highly imbalanced. Again, this might result from confounding, since imbalanced trees contain smaller clades, which are more likely to share a sampling date just by chance (this possibility is supported by simulations showing that unbalanced trees can give reliable results when sampling is balanced; Fig. S3f).

Finally, we have suggested that confounding is likely to be common when serially sampled‐pathogen genomes are used to study the course of a single outbreak. This is partly because confounding can arise naturally from clinical sampling practice. For example, different individuals will often be sampled at different times (Harris *et al*. 2013; Paterson *et al*. 2015), and these individuals will generally contain distinct populations of a pathogen, resulting from transmission barriers between individuals, and population bottlenecks during transmission events. The same also applies to different tissues within an individual (e.g. Sacristán *et al*. 2003; Lee *et al*. 2008; Paterson *et al*. 2015) and to different geographic locations (Holmes 2008). We have also shown that the confounding may be enhanced when little evolutionary change has taken place, which may often be the case during a single outbreak. Consistent with this prediction, we have presented data from an outbreak of *S. aureus* where standard tests provide support for date estimates – and thereby transmission scenarios – that are doubtful on other grounds (Paterson *et al*. 2015).

If confounding of temporal and genetic structures is common, then many dated‐tip analyses may need revisiting. A remarkably common finding in the study of pathogen evolution has been that plausible biogeographic scenarios imply much slower evolutionary rates (and so much older *t*_MRCA_), than are obtained from dated‐tip analyses of serially sampled genomes; often, these estimates differ by several orders of magnitude (Sharp & Simmonds 2011). We have shown that artefactual evidence of temporal signal often leads to false confidence in dates that bear no relation to the true age of divergence (see, e.g. Fig. 1d). As such, results reported here may explain some of the wilder disagreements about pathogen origins.

## Supporting information


**Fig. S1.** The distribution of signed *r* ^2^ values from regressions of phylogenetic root‐to‐tip distance against sampling date, for each of 1000 simulated data sets.
**Fig. S2.** Results of tests of temporal signal for data simulated with a high level of temporal signal, but a ladderised genealogy, in which genetic structure arises over time from the evolution of a single population.
**Fig. S3.** The topologies over which data was simulated.
**Fig. S4.** The MCC tree produced from 157 methicillin‐resistant *S. aureus* genomes from Holden *et al*. (2013).
**Fig. S5.** The Bayesian dating test for *S. aureus* strains sampled from a staff member (34 isolates) during an outbreak in a veterinary hospital (Paterson *et al*.^2015^).
**Fig. S6.** A genealogy of the strains sampled from the dog and the staff member from (Paterson *et al*. 2015).

**Table S1.** Results of tests of temporal signal and dating analyses for simulated data sets with a true t_*MRCA*_ of 10 000 ybp.
**Table S2.** The parameters used to simulate data sets.
**Table S3**. Simulation results when ‘high’ and ‘low’ temporal signal were created by adjusting the substitution rate instead of the sampling dates (see Table S2).
**Table S4**. Tests of temporal signal for an *S. aureus* data set from Holden *et al*. (2013) and subsamples of these data.
**Table S5**. Tests of temporal signal for two *S. aureus* data sets from a single outbreak.

**Appendix S1**. Materials and methods.

**Appendix S2**. A description of the R scripts used to implement a test of confounding temporal and genetic structure and two tests of temporal signal, that can be applied to serially‐sampled sequence data.

**Appendix S3**. An R script that provides functions used in in the tests.

**Appendix S4**. An R script for a regression of phylogenetic root‐to‐tip distance against sampling date with both standard and clustered permutation tests.

**Appendix S5**. An R script for both standard and clustered randomisations of dates over sequences in BEAST xml files.

**Appendix S6**. An R script for a Mantel test of confounding of genetic and temporal distances.

**Appendix S7**. An example sequence data input file.

**Appendix S8**. An example tree input file.

**Appendix S9**. An example BEAST xml input file.
