# Supplementary material for: The effect of genetic structure on molecular dating and tests for temporal signal
Source: Methods Ecol Evol. 2015 Sep 22;7(1):80–9. doi: 10.1111/2041-210X.12466 (PMC4832290; doi:10.1111/2041-210X.12466)
Supplement: Supplementary file 1 — Fig. S1. The distribution of signed r 2 values from regressions of phylogenetic root‐to‐tip distance against sampling date, for each of 1000 simulated data sets. Fig. S2. Results of tests of temporal signal for data simulated with a high level of temporal signal, but a ladderised genealogy, in which genetic structure arises over time from the evolution of a single population. Fig. S3. The topologies over which data was simulated. Fig. S4. The MCC tree produced from 157 methicillin‐resistant S. aureus genomes from Holden et al. (2013). Fig. S5. The Bayesian dating test for S. aureus strains sampled from a staff member (34 isolates) during an outbreak in a veterinary hospital (Paterson et al.2015). Fig. S6. A genealogy of the strains sampled from the dog and the staff member from (Paterson et al. 2015). [file MEE3-7-80-s001.pdf]

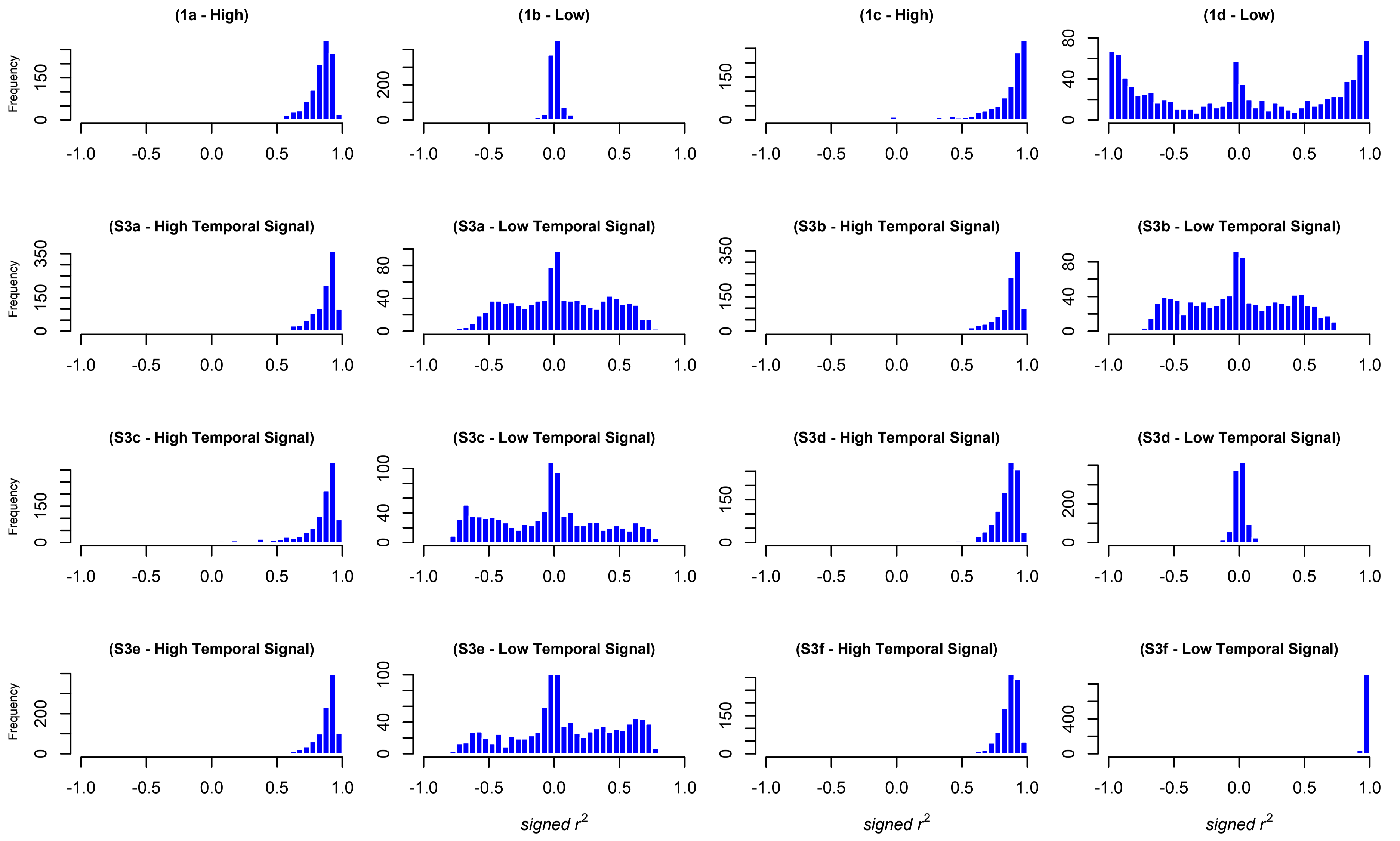

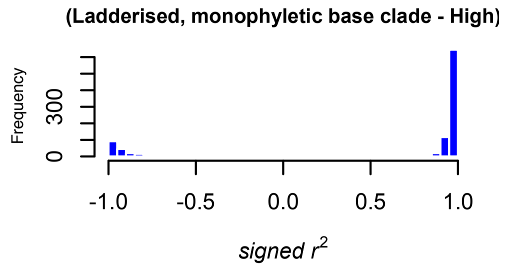

Supplementary Figure S1. The distribution of signed r^2 values from regressions of phylogenetic root-to-tip distance against sampling date, for each of 1000 simulated data sets. Parameters and labels correspond to the trees illustrated in Figures 1, S2 and S3, and results described in Table S1.

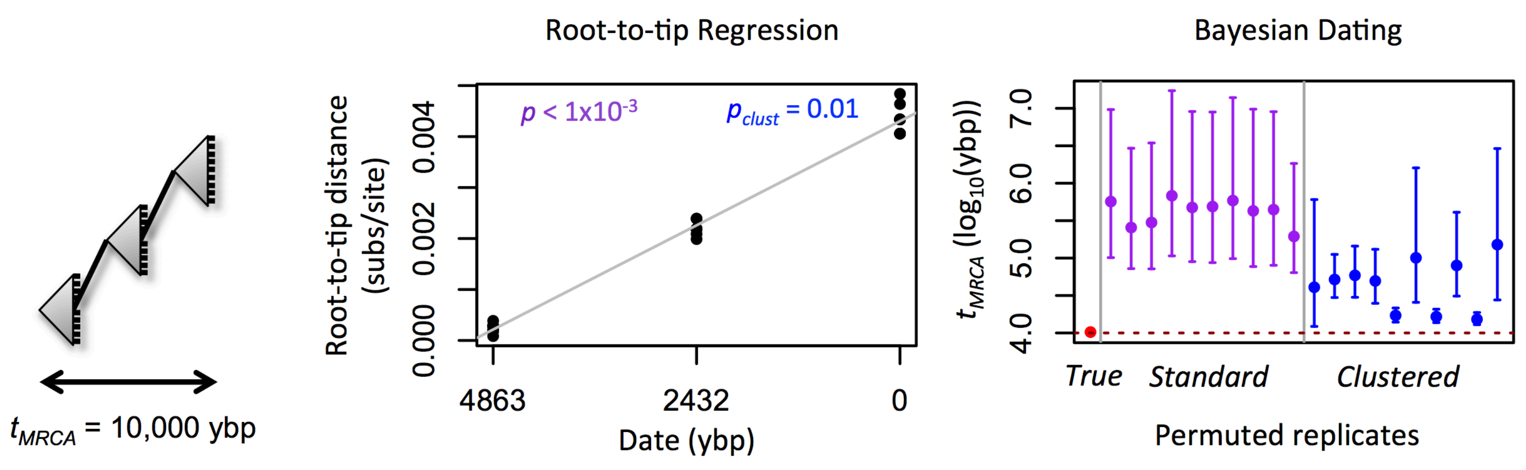

Supplementary Figure S2. Results of tests of temporal signal for data simulated with a high level of temporal signal, but a ladderised genealogy, in which genetic structure arises over time from the evolution of a single population. All other details match Figure 1.

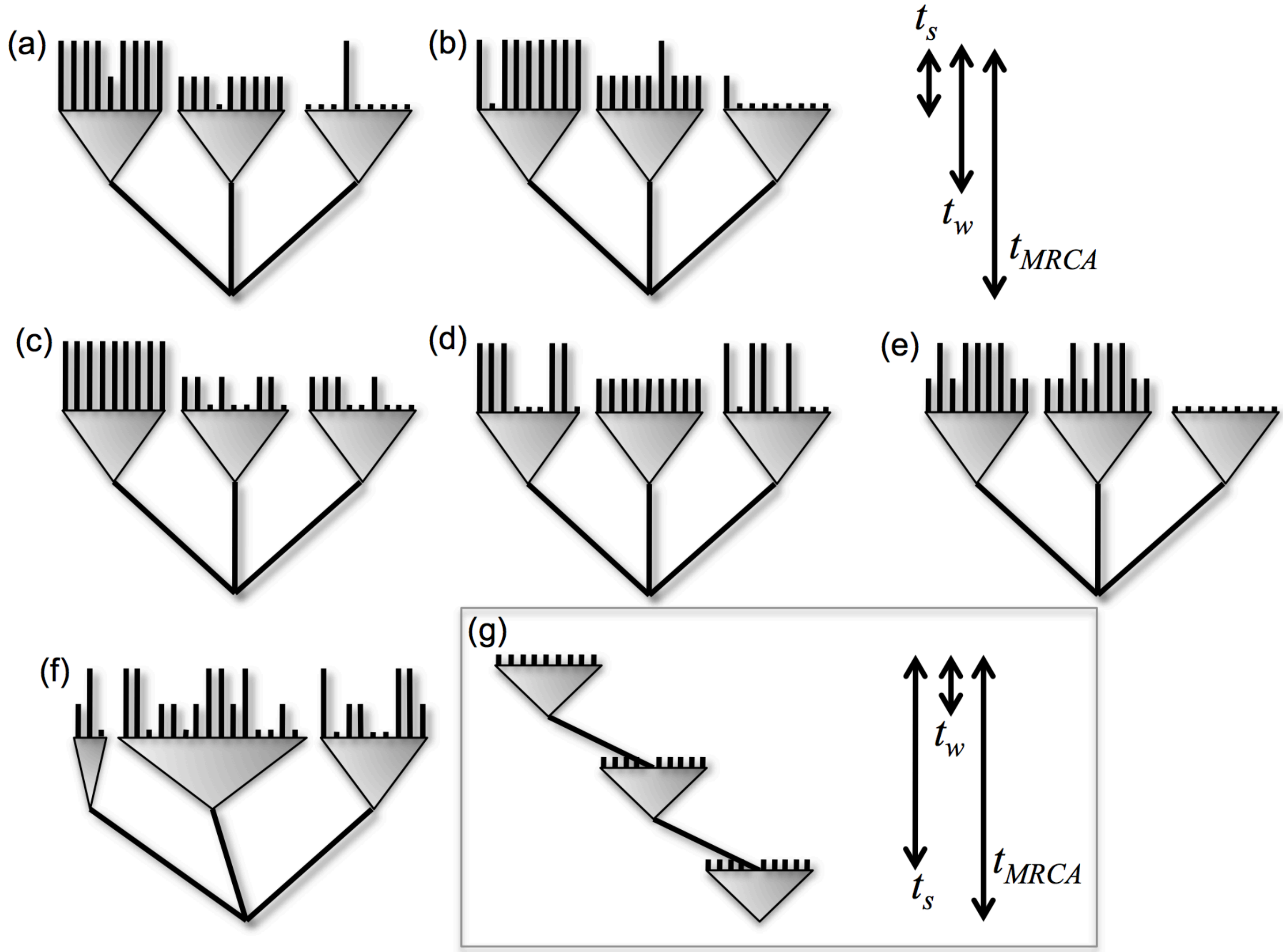

Supplementary Figure S3. Topologies over which data was simulated. (a)-(e) show the five intermediate sampling schemes that were used to produce results in Table S1. For (a) and (b) each clade was sampled 8 times on one sampling date, and once on another sampling date. For (c), (d) and (e) one clade is sampled on a single sampling date, and two are sampled 5 times on one sampling date and 4 times on another sampling date. (f) shows a balanced but unequal sampling of three clades, with one clade sampled 3 times, one 9 times and one 15 times, but with each clade sampled equally at each sampling date. (g) shows the ladderised genealogy in which both temporal and genetic structure arise from the evolution of a single population over time. (b) and (g) also indicate the parameters that describe the simulated tree structures: t_s is the sampling period, t_w is the expected coalescence time for each group of sequences, measured from the most recent sampling time, and t_{MRCA} is the true age of the most recent common ancestor, which was set at 10,000 ybp in all results reported.

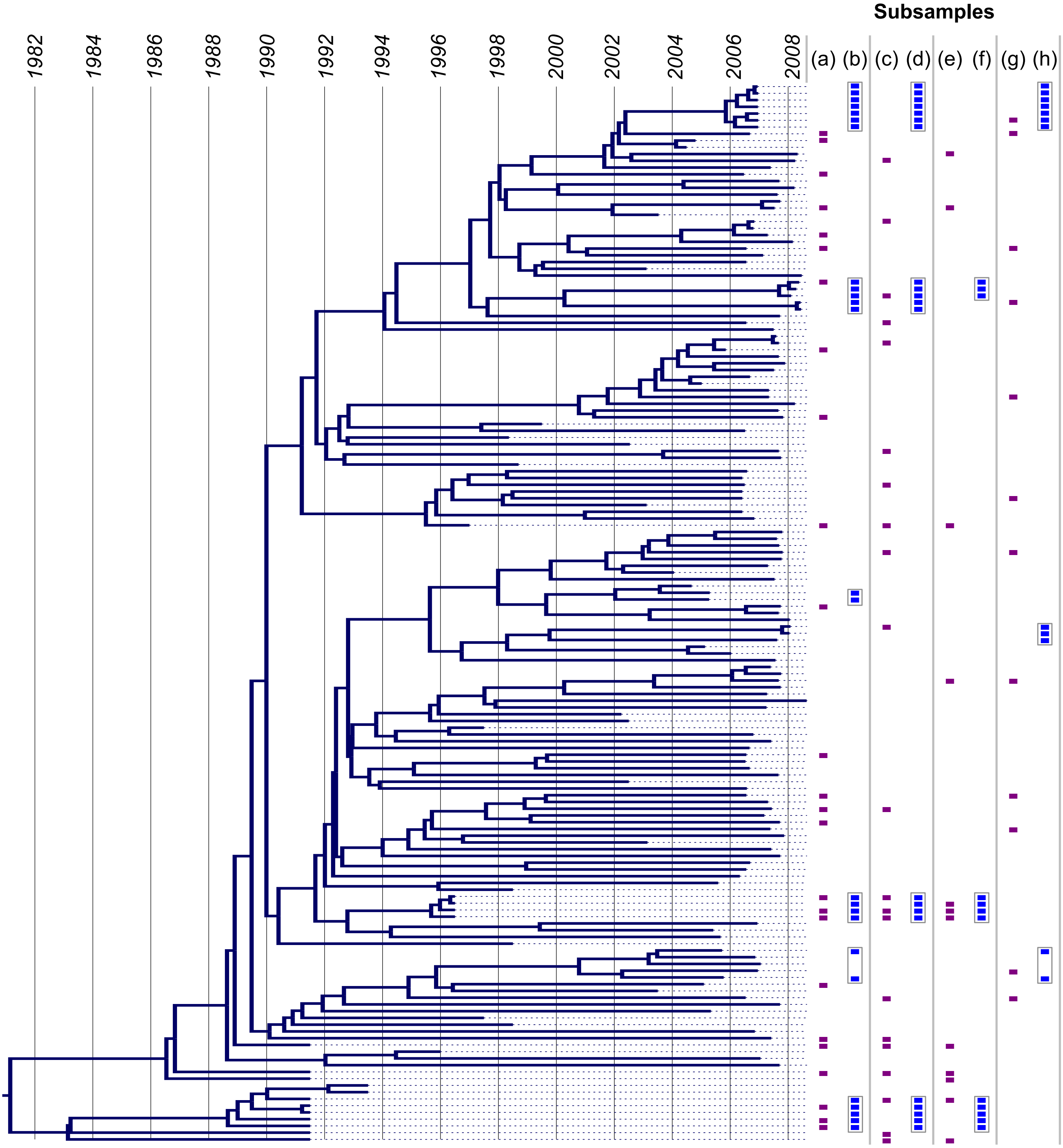

Supplementary Figure S4. The MCC tree produced from 157 methicillin-resistant *S. aureus* genomes from Holden *et al.* (2013). Marks to the right indicate which strains were included in the subsampled data sets, corresponding to results in Figure 3. These subsamples were chosen at random (purple), or in clusters sampled in the same year, to create a confounding of genetic and temporal structure (blue).

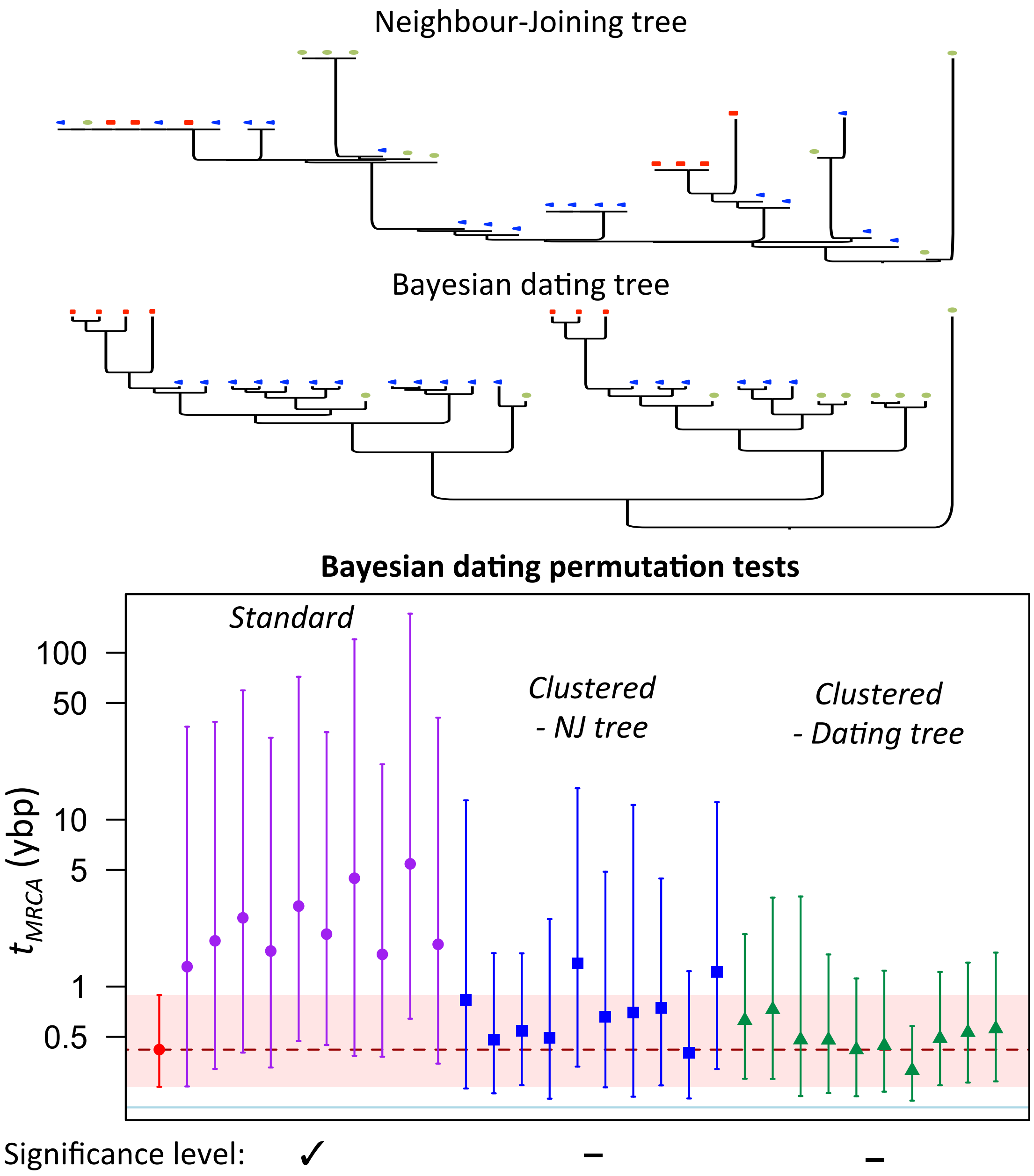

Supplementary Figure S5. The Bayesian dating test for *S. aureus* strains sampled from a staff member (34 isolates) during an outbreak in a veterinary hospital (Paterson *et al.* 2015). Differences in the degree of clustering with sampling date are apparent in the phylogenies of the staff member samples estimated both and without the use of temporal information. Colours and symbol shape represent strains sampled on the same date. The plot shows the MAP estimates of the t_{MRCA}, with 95% HPD Intervals. The t_{MRCA} is reported in years before the most recent sample and is plotted on a log scale. The true estimate (red) is compared to estimates with the sampling dates randomly permuted across sequences (purple), or across single-date monophyletic clusters of sequences identified in a neighbour-joining tree (blue), or across single-date clusters identified from the *BEAST* MCC tree. The blue horizontal line indicates the date of admission of the dog into the veterinary hospital.

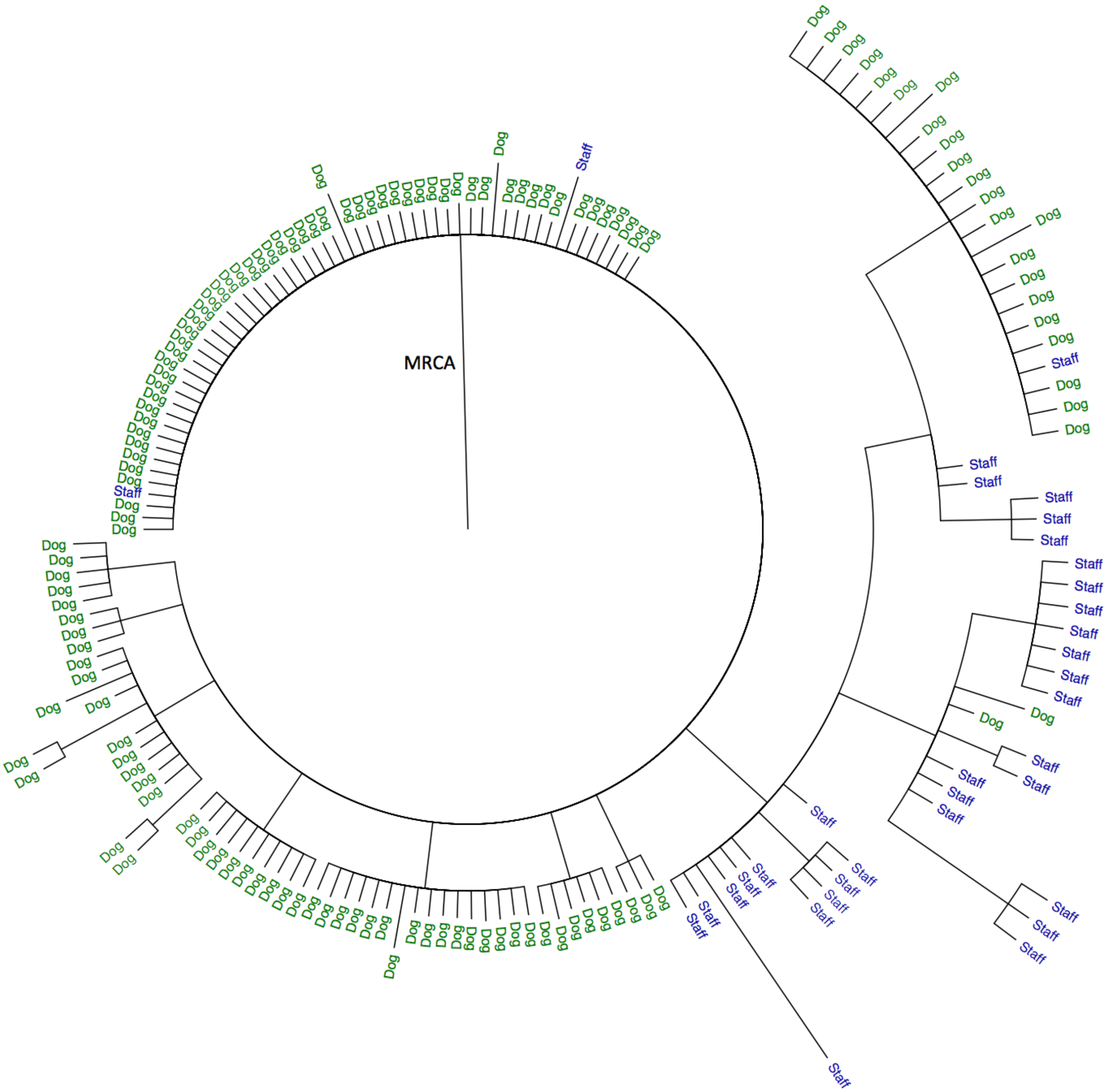

Supplementary Figure S6. A genealogy of the strains sampled from the dog and the staff member from Paterson *et al*. (2015). The genealogy was estimated using *MrBayes* (Huelsenbeck & Ronquist 2001), with a GTR+Γ substitution model. Convergence was assessed using *Tracer* v1.6 (Rambaut & Drummond 2013) and burn-in removed as required. The tree is rooted using an outgroup, as described in (Paterson *et al.* 2015).
