# Supplementary material for: The effect of genetic structure on molecular dating and tests for temporal signal
Source: Methods Ecol Evol. 2015 Sep 22;7(1):80–9. doi: 10.1111/2041-210X.12466 (PMC4832290; doi:10.1111/2041-210X.12466)
Supplement: Supplementary file 2 — Table S1. Results of tests of temporal signal and dating analyses for simulated data sets with a true tMRCA of 10 000 ybp. Table S2. The parameters used to simulate data sets. Table S3. Simulation results when ‘high’ and ‘low’ temporal signal were created by adjusting the substitution rate instead of the sampling dates (see Table S2). Table S4. Tests of temporal signal for an S. aureus data set from Holden et al. (2013) and subsamples of these data. Table S5. Tests of temporal signal for two S. aureus data sets from a single outbreak. [file MEE3-7-80-s002.pdf]

Supplementary Table S1. Results of tests of temporal signal and dating analyses for simulated data sets with a true t_{MRCA} of 10,000 ybp. Data sets were simulated under various sampling conditions, and with different levels of true temporal signal, as illustrated in Figures 1, S2 and S3. For the root-to-tip regression dating analysis, the results show (1) the proportion of 1000 simulated data sets that yielded a high positive correlation between sampling dates and root-to-tip distance ($r > 0.63$). All other results are for two typical replicates, and show (2) the *r* value for that replicate, (3) the *p*-value from a permutation test of significance for the regression (* $p<0.05$; ** $p<0.01$), and (4) the t_{MRCA} estimate, as obtained from the regression intercept. For the Bayesian dating analysis, results show (5) the results of a permutation test of temporal signal asking whether the true date estimate is outlying compared to results with randomised sampling dates. Here, ✓ indicates that the true MAP estimate lay outside of the range of the MAP estimates from the randomised data sets, ✓✓ indicates that the true MAP estimate is not within the HPD intervals of the estimates from randomised data sets, and ✓✓✓ indicates that the HPD interval of the true estimate does not overlap with the HPD intervals of estimates from the randomised data sets. (6) the Maximum *A Posteriori* estimate of the t_{MRCA} from the Bayesian dating analysis, with 95% credible intervals (Highest Posterior Density) is given. Also shown for a subset of simulations is the increase in the *AICM* values for a model that includes sampling dates compared to one that doesn't, and the significance of this value are then given (* $p<0.05$; ** $p<0.01$; see methods). The final columns show results of the two permutation tests after applying the clustering procedure introduced here.

Simulation conditions				Root-to-tip Regression				Bayesian Dating				Clustered permutation	
**Sampling**	**Temporal structure**	**Fig.**		**% high *r***	***r***	**Permutation *p***	**t_{MRCA} (ybp)**	**Permutation test**	**t_{MRCA} MAP (95% CIs)**		**Δ*AICM***	**Regression *p***	**Bayesian Dating**
Balanced	High	(1a)	*i.*	100	0.94	$<1x10^{-3}$ **	9,647	✓✓✓	10,317	(9,164 – 11,645)	277.6 **	$<1x10^{-3}$ **	✓✓✓
			ii.		0.94	$<1x10^{-3}$ **	10,511	✓✓✓	10,795	(9,597 – 12,187)	265.5 **	$<1x10^{-3}$ **	✓✓✓
	Low	(1b)	*i.*	0	0.00	0.38	1,680	✓	2,613	(906 – 17,065)	-4.3	0.42	-
			ii.		0.00	0.40	4,453	-	3,387	(1,057 – 43,312)	-6.3	0.39	-
Confounded	High	(1c)	*i.*	90	0.98	$<1x10^{-3}$ **	8,741	✓✓✓	5,006	(3,762 – 7,012)	-10.7	0.17	✓
			ii.		0.98	$<1x10^{-3}$ **	19,613	✓✓✓	4,609	(3,502 – 6,362)	-6.3	0.33	-

	Low	(1d)	*i.*	36	0.96	<1x10^{-3} **	1,114	✓✓✓	51	(37 – 95)	-13.6		0.17	-
			ii.		0.97	<1x10^{-3} **	178	✓✓✓	64	(38 – 107)	-8.3		0.50	-
Intermediate	High	(S3a)	*i.*	98	0.96	<1x10^{-3} **	12,036	✓✓✓	9,757	(8,232 – 11,863)	101.7	**	<1x10^{-3} **	✓✓✓
			ii.		0.96	<1x10^{-3} **	8,835	✓✓✓	9,245	(7,743 – 11,896)	49.5	**	<1x10^{-3} **	-
	Low		*i.*	22	0.66	<1x10^{-3} **	527	✓✓	2,400	(687 – 30,221)	-3.6		0.03 *	-
			ii.		0.69	<1x10^{-3} **	290	-	1,147	(449 – 5,864)	-0.6		0.03 *	-
Intermediate	High	(S3b)	*i.*	97	0.96	<1x10^{-3} **	12,338	✓✓✓	10,641	(8,571 – 14,140)	-		<1x10^{-3} **	✓
			ii.		0.95	<1x10^{-3} **	9,264	✓✓✓	10,074	(8,606 – 11,887)	-		<1x10^{-3}	✓✓
	Low		*i.*	19	-0.10	0.68	-7,037	-	1,597	(554 – 17,915)	-		0.73	-
			ii.		0.51	0.01 *	782	-	2,209	(700 – 20,490)	-		0.04 *	-
Intermediate	High	(S3c)	*i.*	95	0.87	<1x10^{-3} **	15,076	✓✓✓	8,808	(7,308 – 11,110)	-		0.01 *	✓✓
			ii.		0.95	<1x10^{-3} **	9,264	✓✓✓	11,070	(8,883 – 14,205)	-		<1x10^{-3} **	✓✓
	Low		*i.*	15	0.6	<1x10^{-3} **	348	-	3,214	(713 – 66,299)	-		0.04 *	-
			ii.		-0.10	0.71	-3,076	-	3,472	(746 – 75,254)	-		0.70	-
Intermediate	High	(S3d)	*i.*	100	0.94	<1x10^{-3} **	11,798	✓✓✓	9,316	(8,182 – 10,696)	-		<1x10^{-3} **	✓✓✓
			ii.		0.94	<1x10^{-3} **	10,318	✓✓✓	10,290	(9,022 – 11,768)	-		<1x10^{-3} **	✓✓✓
	Low		*i.*	0	0.00	0.84	-4,390	-	4,755	(1,406 – 70,551)	-		0.99	-

			ii.		0.10	0.61	5,242	-	3,841	(1,468 – 20,391)	-		0.84	-
Intermediate	High	(S3e)	*i.*	99	0.96	<1x10^{-3} **	7,892	✓✓✓	7,944	(6,861 – 9,436)	42.5	**	0.01 *	✓
			ii.		0.96	<1x10^{-3} **	8,601	✓✓✓	9,444	(459 – 11,561)	116.0	**	<1x10^{-3} **	✓
	Low		*i.*	40	0.4	0.02 *	656	✓	1,338	(459 – 11,561)	-6.4		0.12	-
			ii.		0.86	<1x10^{-3} **	199	✓✓	1,359	(484 – 8,432)	-3.2		0.03 *	-
Ladderised	High	(S2, S3g)	*i.*	100	1.00	<1x10^{-3} **	10,237	✓✓✓	10,269	(9,974– 10,868)	37.0	**	0.007 *	✓✓✓
			ii.		1.00	<1x10^{-3} **	9,875	✓✓✓	9,777	(9,343 – 10,465)	-		0.002 *	✓✓✓
Ladderised – monophyletic basal clade	High			80	0.96	<1x10^{-3} **	12,905	✓✓✓	10,237	(9,795 – 10,935)	-		0.065	-
					-0.96	1	-2,248	✓✓✓	10,600	(9,487 – 12,436)	-		1	-
Unequal clades, balanced	High	(S3f)	*i.*	100	0.95	<1x10^{-3} **	9,791	✓✓✓	10,469	(9,326 – 11,923)	-		<1x10^{-3} **	✓✓✓
			ii.		0.95	<1x10^{-3} **	10,754	✓✓✓	10,932	(9,656 – 12,736)	-		<1x10^{-3} **	✓✓✓

Supplementary Table S2. The parameters used to simulate data sets. S = number of nucleotide sites in the genome, μ = rate of molecular evolution, substitutions/site/year, t_s = time period over which strains are sampled, t_w = the expected age of each clade, and t_{MRCA} = the time to most recent common ancestor of the data set.

Simulation conditions								
**Results**	**Temporal Signal**	$S\mu t_s$ (subs/ genome)	$S\mu t_w$ (subs/ genome)	$S\mu t_{MRCA}$ (subs/ genome)	**Tree structure** t_w/t_s	**Number of bases** S	**Substitution rate** μ (subs/site/year)	***Time to MRCA*** t_{MRCA} (ybp)
Table S1; Fig. 1a,c, Fig. S3a-f	High	40	80	160	2	10,000	1.60×10^{-6}	10,000
Table S1; Fig. 1b,d; Fig. S3a-f	Low	0.4	80	160	200	10,000	1.60×10^{-6}	10,000
Table S1; Fig. S2, S3g	High	40	10	50	0.25	10,000	$\sim5.00\times10^{-7}$	10,000
Table S3	High	40	1000	2000	25	10,000	2.00×10^{-5}	10,000
Table S3	Low	0.4	10	20	25	10,000	2.00×10^{-5}	10,000

Supplementary Table S3. Simulation results when “high” and “low” temporal signal were created by adjusting the substitution rate instead of the sampling dates (see Table S2). Genealogies match those illustrated in Fig. 1a,c. All other details match Table S1.

**Simulation conditions**			**Root-to-tip Regression**				**Bayesian Dating**			**Clustered permutation**	
**Sampling**	**Temporal structure**		**% high *r***	***r***	***Permutation p***	t_{MRCA} (ybp)	**Permutation test**	t_{MRCA} MAP (95% CIs)		**Regression *p***	**Bayesian Dating**
Balanced	High	*i.*	29	0.10	0.34	8,584	✓✓	9,808	(8,122 – 12,161)	0.35	✓✓✓
		ii.		0.00	0.43	4,530	✓✓	12,657	(10,196 – 16,208)	0.48	✓✓
	Low	*i.*	0	0.10	0.35	6,109	-	18,007	(3,148 – 437,729)	0.35	-
		ii.		0.00	0.43	11,179	-	7,848	(1,706 – 3,148)	0.42	-
Confounded	High	*i.*	48	-0.14	0.74	-6,561	✓✓✓	1,044	(693 – 2,679)	0.67	-
		ii.		0.96	$<1x10^{-3}$ **	2,803	✓✓✓	1,622	(855 – 3,456)	0.17	✓
	Low	*i.*	36	0.96	$<1x10^{-3}$ **	417	✓✓✓	216	(204 – 279)	0.17	-
		ii.		0.96	$<1x10^{-3}$ **	328	✓✓✓	218	(206 – 269)	0.17	✓

Supplementary Table S4. Tests of temporal signal for the *S. aureus* data set from Holden *et al*. (2013) and subsamples of these data. The duration of the sampling period, number of strains and number of single-date clusters are given for each data set. Details of all tests match those shown in Table S1, but we also add the Maximum *A Posteriori* estimate of the mean evolutionary rate, with 95% credible intervals (Highest Posterior Density), and Bayes factor results, comparing the fit of models with and without the sampling dates included. Bayes factors support for the inclusion of sampling dates is labelled as * "positive"; ** "decisive" (Kass & Rafftery 1995).

Data set			Root-to-tip Regression			Bayesian Dating					Clustered permutation	
**Sampling period** (years)	**no. strains**	**no. clusters**	***r***	***Permutation p***	t_{MRCA} (ybp)	**Permutation test**	t_{MRCA} MAP (95% CIs)	***Mean rate*** (subs/site/year x10^{-6})	**Δ*AICM***	***Bayes factor***	**Regression *p***	**Bayesian Dating**
17	158	116	0.76	$<1\times10^{-3}$ **	29	✓✓✓	27.68 (24.57 – 31.13)	1.34 (1.22 – 1.47)	34.4 **	-	$<1\times10^{-3}$ **	✓✓✓
17	25	21	0.82	$<1\times10^{-3}$ **	30	✓✓	28.07 (23.40 – 36.40)	1.19 (0.92 – 1.46)	1.0	1×10^{6} **	$<1\times10^{-3}$ **	✓✓
17	25	6	0.94	$<1\times10^{-3}$ **	37	✓✓✓	26.10 (17.9 – 33.5)	1.28 (0.72 – 2.08)	1.3	5×10^{223} **	0.089	-
17	21	17	0.84	$<1\times10^{-3}$ **	28	✓	27.59 (23.22 – 35.84)	1.21 (0.93 – 1.48)	-5.9	4×10^{5} **	$<1\times10^{-3}$ **	✓
17	21	4	0.98	$<1\times10^{-3}$ **	35	✓✓✓	18.60 (17.0 – 22.4)	2.91 (1.31 – 4.11)	0.6	2×10^{4} **	0.08	-
17	12	9	0.92	$<1\times10^{-3}$ **	30	✓	26.34 (21.36 – 33.79)	1.26 (0.88 – 1.74)	-5.6	2×10^{7} **	0.004 **	✓
17	12	3	0.95	$<1\times10^{-3}$ **	41	✓✓✓	18.40 (16.9 – 20.2)	2.66 (1.62 – 3.67)	6.7 *	206 **	0.57	-
3	12	11	-0.00	0.49	-273	-	8.88 (2.43 – 149.79)	0.88 (0.00 – 7.42)	-538.6	0.29	0.49	-
3	12	3	0.99	$<1\times10^{-3}$ **	6	✓✓✓	2.80 (2.38 – 3.65)	13.8 (6.56 – 21.7)	-5.1	3.43 *	0.29	-

Supplementary Table S5. Tests of temporal signal for two *S. aureus* data sets from a single outbreak (Paterson *et al.* 2015). The duration of the sampling period, number of strains and number of single-date clusters are given for each data set. The details of the tests reported match Table S4.

Data set			Root-to-tip Regression			Bayesian Dating					Mantel test (*p*)		
**Individual**	**Sampling period** (days)	**no. strains**	***r***	***Permutation p***	t_{MRCA} (ybp)	**Permutation test**	t_{MRCA} MAP (95% CIs)		***Substitution rate*** (subs/site/year x10^6) MAP (95% CIs)		***NJ* tree**	***BEAST* tree**	**Clustered *BEAST***
Dog	7	137	0.00	0.22	0.78	✓✓	0.19	(0.18 – 0.25)	37.7	(8.70 – 76.4)	0.88	<1x10^{-4}	0.09
Staff member	57	34	0.10	0.28	0.98	✓	0.42	(0.25– 0.89)	3.84	(1.30 – 6.75)	0.08	<1x10^{-4}	0.07

			Clustered permutation		
**Individual**	**no. clusters in NJ tree**	***no. clusters in BEAST tree***	**Regression *p* using NJ tree clusters**	**Bayesian Dating using NJ tree clusters**	**Bayesian Dating using *BEAST* tree clusters**
Dog	77	6	0.34	✓	-
Staff member	24	13	0.30	-	-
