# Supplementary material for: The effect of genetic structure on molecular dating and tests for temporal signal
Source: Methods Ecol Evol. 2015 Sep 22;7(1):80–9. doi: 10.1111/2041-210X.12466 (PMC4832290; doi:10.1111/2041-210X.12466)
Supplement: Supplementary file 3 — Appendix S1. Materials and methods. [file MEE3-7-80-s003.pdf]

Supplementary Methods

Simulation of topologies

We simulated phylogenies with 27 sequences, each of which was assumed to have been sampled on one of three equally spaced sampling dates. To simulate structured populations (Fig. 1a-d), we assumed three monophyletic clades of equal size, with a polytomous root (such as may commonly arise from the action of natural selection, or drift within a structured population; Barton *et al.* 2010). We then varied the sampling strategy by changing the distribution of sampling dates over these three clades. The topology of each clade was simulated under a standard coalescent process, with variation in sampling time modelled through the addition of tips at different points during the coalescence. To simulate ladderised genealogies (Supplementary Fig. S2) we assumed a single clade, with a rate of coalescence that was rapid compared to the gaps between sampling dates.

Simulation of molecular evolution

The evolution of DNA sequences over each topology was simulated using *Seq-Gen* (Rambaut & Grassly 1997). We simulated a genome of 10,000 bp, with a HKY model of nucleotide substitution with no variation in rates over sites, and a base composition comparable to the *S. aureus* data (34% A, 34% T, 16% G and 16% C). While the simulated sequences are much smaller than bacterial or some viral genomes (Belshaw *et al.* 2008), we ensured that the rates and evolutionary time-scales used meant that no saturation of sites would occur, and therefore that genome length would not affect our results. We note that this model is larger than the model used to estimate branch lengths for the regression test. However, examination of the K80 branch lengths suggested that they were generally close to the true values. More importantly, underparameterisation cannot explain the different outcomes for the different data sets shown in Figure 1.

For the structured populations (Fig. 1), we set branch lengths such that an expected 80 substitutions per genome would accrue from the most recent sampling date to the base of each of the three clades, and a further expected 80 substitutions from the base of each clade to the root of the genealogy. To model low temporal structure, we chose sampling dates such that only 0.4 substitutions per genome were expected to accrue during the sampling period. This was increased to 40 substitutions per genome to model high temporal structure. To simulate ladderised genealogies (Supplementary Figs. S2-S3) we kept the same level of high temporal structure, assuming an expected 40 substitutions per genome during the sampling period. We then set the rate of coalescence, such that 10 substitutions per genome were expected before the coalescence of each set of 9 sequences sampled on a single date.

For the dating analysis, we set the root of all trees at 10,000 years before the present (ybp), which implied a substitution rate of (80+80)/10,000bp/10,000ybp = 1.6×10^{-6} subs/site/year for the structured populations and an expected (40+10)/10,000bp/10,000ybp = 5.0×10^{-7} subs/site/year for the ladderised genealogies. Full details of parameters are given in Supplementary Table S2 and Figure S3. 1000 topologies and sets of sequences were simulated under each set of parameters. Some analyses used all 1000 data sets, while others used two "typical" data sets. These data sets were chosen on the basis of results from regression analysis (see below), and were the data sets whose correlation coefficients, r, were closest to the positive mode of the distribution of r values inferred from the 1000 replicate data sets, choosing the roots so as to maximise this quantity.

Alternative tree topologies

Results in the main text vary the level of true temporal structure by adjusting the sampling dates, while keeping the rest of the parameters unchanged, but we also explored the alternative approach, of keeping the topology unchanged, but adjusting the rate of nucleotide substitution. Full details of these simulations can be found in Supplementary Tables S2-S3.

Reanalysis of published data

To reanalyse the data of Holden *et al.* (2013), we used their alignments of the core genome after the removal of poor quality sites and strains. We additionally excluded 5 basal strains that were not methicillin resistant, in order to retain the same t_{MRCA} for the subsampled data sets. For our analysis of the data of Paterson *et al.* (2015) we used their cleaned alignments of the core genome, but retained only strains sampled from the "index dog" and the related "clade 1" strains from "staff member A".

Supplemental References

Barton, N.H., Kelleher, J. & Etheridge, A.M. (2010). A new model for extinction and recolonization in two dimensions: quantifying phylogeography. *Evolution*, **64**, 2701–2715.

Belshaw, R., Gardner, A., Rambaut, A. & Pybus, O.G. (2008). Pacing a small cage: mutation and RNA viruses. *Trends in Ecology & Evolution*, **23**, 188–193.

Rambaut, A. & Grassly, N.C. (1997). Seq-Gen: an application for the Monte Carlo simulation of DNA sequence evolution along phylogenetic trees. *Computer applications in the biosciences*, **13**, 235–238.
