# Supplementary material for: The effect of genetic structure on molecular dating and tests for temporal signal
Source: Methods Ecol Evol. 2015 Sep 22;7(1):80–9. doi: 10.1111/2041-210X.12466 (PMC4832290; doi:10.1111/2041-210X.12466)
Supplement: Supplementary file 4 — Appendix S2. A description of the R scripts used to implement a test of confounding temporal and genetic structure and two tests of temporal signal, that can be applied to serially‐sampled sequence data. [file MEE3-7-80-s004.pdf]

R scripts are provided that implement a test of confounding of temporal and genetic structure and two tests of temporal signal, both standard and clustered versions, that can be applied to serially-sampled sequence data.

1. A Mantel test of confounding of genetic and temporal distances.

Functions to apply this test are provided in **mantel.confounding.R** and **tempsignalfunctions.R**.

The function to run the regression and permutation test is

mantel.confounding.test(inputfile, datatype='dna.fasta', dateschar='_', dates=NULL, auto.round.dates=F, rounded.dates=NULL, nreps=1000, pval=0.05, use.clusters=F, clusters=NULL, do.plot=F, reroot=T, stat='nve-rms')

Arguments *(defaults)*:

inputfile	The name of the file that contains the sequence alignment or tree from which pairwise genetic distances will be calculated. This can be an alignment in fasta format, a tree file in nexus format or a tree file in newick format.
datatype *('dna.fasta')*	The type of data in the input file. It can be changed to 'tree.nexus' or 'tree.newick' if inputting a tree.
dateschar *('_')*	If dates are not otherwise specified, the sampling dates that are used to calculate temporal distances are extracted from the sequence names or tip labels. They must be the last element of the name, after a character specified by 'dateschar'. They must be in numeric format.
dates *(NULL)*	When specified, this overrides the reading of dates from sequence names or tip labels. It should provide dates in numeric format (e.g. c(1,2,3)), in the same order as the tip labels of the tree (phy$tip.label) or as the sequences in the alignment.
auto.round.dates *(F)*	Set to true you want your dates to be rounded to zero decimal places for the description of 'single-date' clusters (non-rounded dates will be used in the test).
rounded.dates *(NULL)*	A list of rounded dates in numeric format in the same order as the tip labels of the tree (phy$tip.label) can be provided to be used in defining single-date clusters. These 'rounded.dates', if specified, will be used when single-dates clusters are defined. The values provided in 'dates' will be used in the test.
nreps *(1000)*	The number of randomised data sets used to estimate a *p*-value.
pval *(0.05)*	This is the *p*-value cut-off for the test.
use.clusters *(F)*	Set to true for the clustered test.
clusters *(NULL)*	A vector of consecutive integers starting from one, e.g. c(1,1, 2,3), in numeric format, in the same order as the tip labels of the tree or sequences in the alignment, can be proved to define clusters. If specified, these clusters will be used instead of defining clusters as

	single-date clades. The vector should describe, for each sequence, which cluster it belongs to, e.g. c(1, 1, 2, 3) describes data with 3 clusters, with the first two samples in the data in the same cluster.
do.plot *(T)*	Set to false if you don't want a plot of the test to be shown.
reroot *(T)*	Set to false if you are using a rooted phylogeny and don't to change the root when clusters are being chosen.
stat *('nve-rms')*	Can be set to (1)'nve-rms', (2)'r2' or (3)'r' depending on whether you want the root for clustering to be chosen so as to (1) minimise the residual mean squares of the model, (2) maximise r^2 or (3) maximise r. We recommend (1).

Libraries required: **ape**, **ade4** and **seqinR** (Paradis, E., Claude, J. & Strimmer, K. (2004). APE: Analyses of phylogenetics and evolution in R language. *Bioinformatics*, 20, 289–290; Dray, S. & Dufour, A.B. (2007). The ade4 package: implementing the duality diagram for ecologists. *Journal of Statistical Software*, 22, 1-20; Charif, D. & Lobry, J.R. (2007). SeqinR 1.0-2: a contributed package to the R project for statistical computing devoted to biological sequences retrieval and analysis. Structural approaches to sequence evolution: Molecules, networks, populations. Editors: U. Bastolla, M. Porto, H.E. Roman & M. Vendruscolo. Springer Verlag, New York.).

This function applies a test for confounding between genetic and temporal distance in a serially sampled data set. It can be applied to a nucleotide sequence alignment or a tree estimated from the sequence data. Dates can be provided as the last element of the sequence or tip name, or independently, as an argument of the function. The function applies a Mantel test to the genetic (either pairwise or patristic) and temporal distances, between samples or clusters of samples, to determine whether they are confounded. If there is evidence of confounding in a data set, the function can also be used to establish whether a particular clustering of the data is sufficient to eliminate confounding. The function can cluster data into single-date clades (using either the dates provided, the dates provided, rounded to zero decimal places, or a specified rounding of the dates), or can use a user-specified clustering of the data. Unless otherwise specified, when defining clusters, the function will re-root the tree estimated from or provided in the input data so as to maximise the fit of a regression of root-to-tip distance against sampling date. A *p*-value and a plot, which shows the relationship between temporal and genetic distance for individual sequences, or clusters of sequences, are returned. When a clustered test is performed, a plot is also returned that represents how the data was clustered, through the colouring of the tips of a tree.

2. A regression of phylogenetic root-to-tip distance against sampling date with a permutation test to determine a *p*-value for the correlation.

Functions to apply this test are provided in **rand.regression.R** and **tempsignalfunctions.R**.

The function to run the regression and permutation test is

pathogen.permutation.test(phy, dates, auto.round.dates=F, rounded.dates=NULL, reroot=T, stat='nve-rms', nreps=1000, use.clusters=T, clusters=NULL, print.progress=T, output.rooted.tree=T)

Arguments *(defaults)*:

phy	A phylogeny estimated without the use of dates (e.g. a NJ tree).
dates	A list of dates are required in numeric format (e.g. c(1,2,3)) in the same order as the tip labels of the tree (phy$tip.label). Dates should be specified as a number of time units since some time in the past.
auto.round.dates *(F)*	Set to true you want your dates to be rounded to zero decimal places for the definition of 'single-date' clusters. Non-rounded dates will be used in the regression, and for permuted analyses the non-rounded dates from a 'single-date' clade will be randomly sampled with replacement when assigning dates to another 'single-date' clade.
rounded.dates *(NULL)*	A list of rounded dates in numeric format in the same order as the tip labels of the tree (phy$tip.label) can be proved to be used in defining single-date clusters. These 'rounded.dates', if specified, will be used when single-dates clusters are defined. Again, the values provided in 'dates' will be used in the regression.
reroot *(T)*	Set to false if you are using a rooted phylogeny and don't to change the root to maximise the model fit/correlation of the regression.
stat *('nve-rms')*	Can be set to (1)'nve-rms', (2)'r2' or (3)'r' depending on whether you want the root to be chosen so as to (1) minimise the residual mean squares of the model, (2) maximise r^2 or (3) maximise *r*. We recommend (1).
nreps *(1000)*	The number of randomised data sets you want to create to estimate a *p*-value (this will be limited by the number of possible permutations).
use.clusters *(T)*	Set to false if you want to run a standard, rather than clustered, permutation.
clusters *(NULL)*	A list of consecutive integers starting from one, e.g. c(1,1, 2,3), in numeric format, in the same order as the tip labels of the tree (phy$tip.label), can be proved to define clusters. If specified, these clusters will be used instead of defining clusters as single-date clades. The vector should describe, for each sequence, which cluster it belongs to, e.g. c(1, 1, 2, 3) describes data with 3 clusters, with the first two samples in the data in the same cluster.

print.progress *(T)*	Set to false if you want to suppress the progress statement on the randomisation.
output.rooted.tree *(T)*	Set to false if you don't want the rooted phylogeny to be written to file.

Library required: **ape** (Paradis, E., Claude, J. & Strimmer, K. (2004). APE: Analyses of phylogenetics and evolution in R language. *Bioinformatics,* 20, 289–290).

This test can be performed on a rooted, fully segregating tree (an example of a suitable tree is provided), or the tree can be rooted so as to (1) minimise the residual mean squares of the model, (2) maximise r-squared or (3) maximise signed r-squared. We recommend (1). The function estimates the correlation coefficient, *r,* of a regression of sampling dates against root-to-tip distances, and estimates a *p*-value for this correlation through comparing the true *r*-value to the *r*-value estimated for the data with dates randomly permuted over the tips of the phylogeny. The permutation can be performed over all strains or over clusters of strains. Clusters can be defined as either (i) clades that share the same sampling date (with the phylogeny rooted to maximise the fit of the regression), (ii) clades that share the same date after rounding the dates to zero decimal places, (iii) clades that share the same date when the dates are rounded to a degree specified as an argument of the function ('rounded.dates'), or (iv) sets of sequences specified as an argument of the function ('clusters'). Dates should be provided in numeric format and in the same order as the tip labels of the tree. A function is provided to extract dates from the tip labels of the input tree if the numeric date value is provided after the last '_' in the tip names. The output of the function is an R plot of the regression, a plot describing how the data was clustered (if done), a histogram of the *r*-values of the permuted data (with a vertical red line representing the value of the true ordering), an object that contains details of the test input and results, and the tree rooted so as to maximise model fit written to file.

3. Randomisation of dates over sequences in BEAST xml files.

Functions to apply this test are provided in **rand.xmls.R** and **tempsignalfunctions.R**.

The function to create the xml files with permuted date values is

create.randomised.xmls(xml.file.name, nreps=10, use.clusters=T, do.plot=T, auto.round.dates=F, rounded.dates=NULL, clusters=NULL, stat='nve-rms')

Arguments *(defaults)*:

xml.file.name	A BEAST v1.8 format xml file with dated sequences, dated so that earlier dates have a lower numerical value, i.e. direction = 'forwards'.
nreps *(10)*	Number of randomised xmls to be created (this will be limited by the number of possible permutations).
use.clusters *(T)*	Set to false if you want to do a standard, rather than clustered, permutation.
do.plot *(T)*	Set to false if you don't want a plot of the clusters to be shown.
auto.round.dates *(F)*	Set to true you want your dates to be rounded to zero decimal places for the definition of 'single-date' clusters. Non-rounded dates from a 'single-date' clade will be randomly sampled with replacement when assigning dates to another 'single-date' clade.
rounded.dates *(NULL)*	A list of rounded dates in numeric format in the same order as the sequences in the xml can be proved to be used in defining single-date clusters. These 'rounded.dates', if specified, will be used when single-dates clusters are defined. Again, the values provided in 'dates' will be used in the xmls.
clusters *(NULL)*	A list of consecutive integers starting from one, e.g. c(1,1, 2,3), in numeric format, in the same order as the sequences of the xml, can be proved to define clusters. If specified, these clusters will be used instead of defining clusters as single-date clades. The vector should describe, for each sequence, which cluster it belongs to, e.g. c(1, 1, 2, 3) describes data with 3 clusters, with the first two samples in the data in the same cluster.
stat *('nve-rms')*	Can be set to (1)'nve-rms', (2)'r2' or (3)'r' depending on whether you want the root used for clustering to be chosen so as to (1) minimise the residual mean squares of the model, (2) maximise r^2 or (3) maximise r. We recommend (1).

Library required: **ape** (Paradis, E., Claude, J. & Strimmer, K. (2004). APE: Analyses of phylogenetics and evolution in R language. *Bioinformatics,* 20, 289–290).

The function takes a BEAST v1 xml file as its input and outputs a specified number of replicates with dates permuted over sequences. An example of a suitable input file is provided. The permutation can be performed over all strains or over clusters of strains. Clusters can be defined as either (i) clades that share the same sampling date (with a tree estimated from the

sequence data in the xml, and rooted to maximise the fit of a regression of sampling date against root-to-tip distance), (ii) clades that share the same date after rounding the dates to zero decimal places, (iii) clades that share the same date when the dates are rounded to a degree specified as an argument of the function ('rounded.dates'), or (iv) sets of sequences specified as an argument of the function ('clusters'). The outputted BEAST xml files should be run in BEAST v1, and the estimates of dates or rates compared to the results from the true ordering of dates.

The example input files for these functions are taken from data described in (Holden, M.T.G., Hsu, L.-Y., Kurt, K., Weinert, L.A., Mather, A.E., Harris, S.R., Strommenger, B., Layer, F., Witte, W., de Lencastre, H. *et al*. (2013). A genomic portrait of the emergence, evolution, and global spread of a methicillin-resistant *Staphylococcus aureus* pandemic. *Genome Research.* **23**, 653–664).
